# Hepatitis B knowledge, attitudes and practices among health-care workers in Vanuatu, 2024

**DOI:** 10.5365/wpsar.2026.17.2.1209

**Published:** 2026-04-08

**Authors:** Leila Bell, Kaylene Kalmos, Annie Taissets, Florita Toa, Sereana Natuman, Mark Stoové, Margaret Hellard, Nicole Allard, Caroline van Gemert

**Affiliations:** a Burnet Institute, Melbourne, Victoria, Australia.; b School of Public Health and Preventative Medicine, Monash University, Melbourne, Victoria, Australia.; c Ministry of Health, Port Vila, Vanuatu.; d Vila Central Hospital, Port Vila, Vanuatu.; e Doherty Institute and School of Population and Global Health, University of Melbourne, Melbourne, Victoria, Australia.; f Department of Infectious Diseases, The Alfred Hospital, Melbourne, Victoria, Australia.; g WHO Collaborating Centre for Viral Hepatitis, Victorian Infectious Diseases Reference Laboratory, Royal Melbourne Hospital, at the Peter Doherty Institute for Infection and Immunity, Melbourne, Victoria, Australia.; h Department of Infectious Diseases, University of Melbourne, Melbourne, Victoria, Australia.

## Abstract

**Objective:**

The prevalence of chronic hepatitis B in Vanuatu is high, at approximately 9%. While immunization has been available for infants since 1989, subsets of the adult population remain susceptible, including health-care workers. Prior to a planned roll-out of hepatitis B vaccination for health-care workers, we conducted a knowledge, attitudes and practices survey to inform education programmes aimed at promoting vaccine uptake.

**Methods:**

Clinical and non-clinical health-care professionals at risk of occupational exposure to hepatitis B were invited to complete an online survey from April to June 2024. The survey sought information on hepatitis B knowledge (10 questions), attitudes (6 questions) and clinical practices (3 questions), as well as participant demographics. Participant knowledge scores were calculated and potential associations with demographic factors explored using Fisher’s exact test.

**Results:**

Most of the 50 respondents were female (73%) and worked in either hospitals or the Ministry of Health (82%). Knowledge was high, with a median score of 9 (range: 3–10); 21 participants scored 100%. The proportion of incorrect responses was highest for questions related to treatment availability and transmission risks. We found no evidence of associations between demographic factors and knowledge scores. Most participants believed that hepatitis B vaccines were useful (88%) and prevention and control measures would protect them from infection (96%).

**Discussion:**

Our survey revealed high levels of knowledge and generally positive attitudes towards people with hepatitis B and infection control practices. While our respondents are unlikely to be representative of all health-care workers in Vanuatu, findings offer useful insights into specific knowledge gaps that could be addressed in planned health-care worker education sessions ahead of the vaccination roll-out.

Chronic hepatitis B is a significant burden in Vanuatu, with an average prevalence of approximately 9%. (*1*) Prevalence is higher among people aged 30 years and over; in this age group, estimated prevalence is  14–20%, compared to 3–7% in those under 30 years. (*1*) This difference is likely due to the introduction of hepatitis B immunization in 1989–1990, when a birth dose was added to the routine childhood immunization schedule. (*2*)

The current immunization schedule comprises a hepatitis B birth dose (vaccination within 24 hours of birth) and three additional doses at 6, 10 and 14 weeks as part of the pentavalent vaccine. (*2*, *3*) However, despite the long-standing availability of the hepatitis B vaccine in Vanuatu and international partner support, coverage remains suboptimal in some provinces. (*4*) For example, in 2022, a timely birth dose was given to between 23% and 90% of newborn infants, while third-dose coverage ranged between 41% and 79% depending on the province (unpublished programmatic data from the Ministry of Health, Vanuatu). At present, Vanuatu does not have an adult vaccination programme, including for adults with occupational risk factors such as health-care workers (HCWs). Recent seroprevalence data are sparse, but given the history of variable childhood vaccination rates, there is likely a large number of susceptible adults in the population. (*4*-*7*)

Occupational risk factors for hepatitis B infection among HCWs are well documented globally and arise due to potential exposure to infected blood or body fluids. (*8*, *9*) However, few studies on occupational risk factors and hepatitis B infection in health facilities have been conducted in the Pacific region. (*10*) While some HCWs in Vanuatu may have been vaccinated while training abroad, this group probably only forms a small proportion of the workforce. It is more likely that a significant proportion of HCWs in Vanuatu are unvaccinated as they were born before the introduction of routine childhood immunization in 1989, and there is no requirement for HCWs in the country to be vaccinated. Additionally, while infection prevention and control (IPC) guidelines exist, adherence levels to recommended practices are not known.

In line with World Health Organization (WHO) recommendations, and because of high prevalence, low vaccine coverage among older people and occupational risks, Vanuatu’s National HIV, STI and Viral Hepatitis Committee has declared hepatitis B vaccination of HCWs and people working in health-care settings a priority. (*11*) Roll-out of a programme to vaccinate the workforce was scheduled to start in 2025. Previously documented vaccine hesitancy among HCWs during the COVID-19 pandemic, both in Vanuatu and globally, (*12*, *13*) prompted the Committee to undertake an exploratory study to assess baseline knowledge, attitudes and practices around hepatitis B vaccination among HCWs before the roll-out. In addition, numerous studies have demonstrated that higher levels of health knowledge are associated with positive health behaviours, including vaccine uptake. (*14*, *15*) Thus, a key objective of the study was to identify and better understand barriers to vaccine uptake among HCWs, and to use the data collected to inform targeted vaccination and education programmes.

## Methods

### Target population and recruitment

The target population was HCWs at health facilities in Vanuatu, specifically health-care professionals working in a health facility or anyone in a public health role who was at increased risk of hepatitis B infection due to occupational exposure to blood or body fluids. The target population thus included doctors, nurses, midwives, laboratory officers and technicians, dentists, dental technicians, and other allied health professionals including public health officers and drivers, as well as janitorial and administrative staff.

The survey was distributed via e-mail and known online HCW chat groups. All HCWs working in public and private health facilities in Vanuatu at the time of the survey were eligible to participate. Students, interns and volunteers were also eligible to participate. The estimated size of the health workforce in Vanuatu was approximately 1000 people, (*16*) of whom around 150 were contacted via e-mail; an unknown number of online chat group members were invited to complete the survey.

### Study design and data collection

**Fig. 1:**
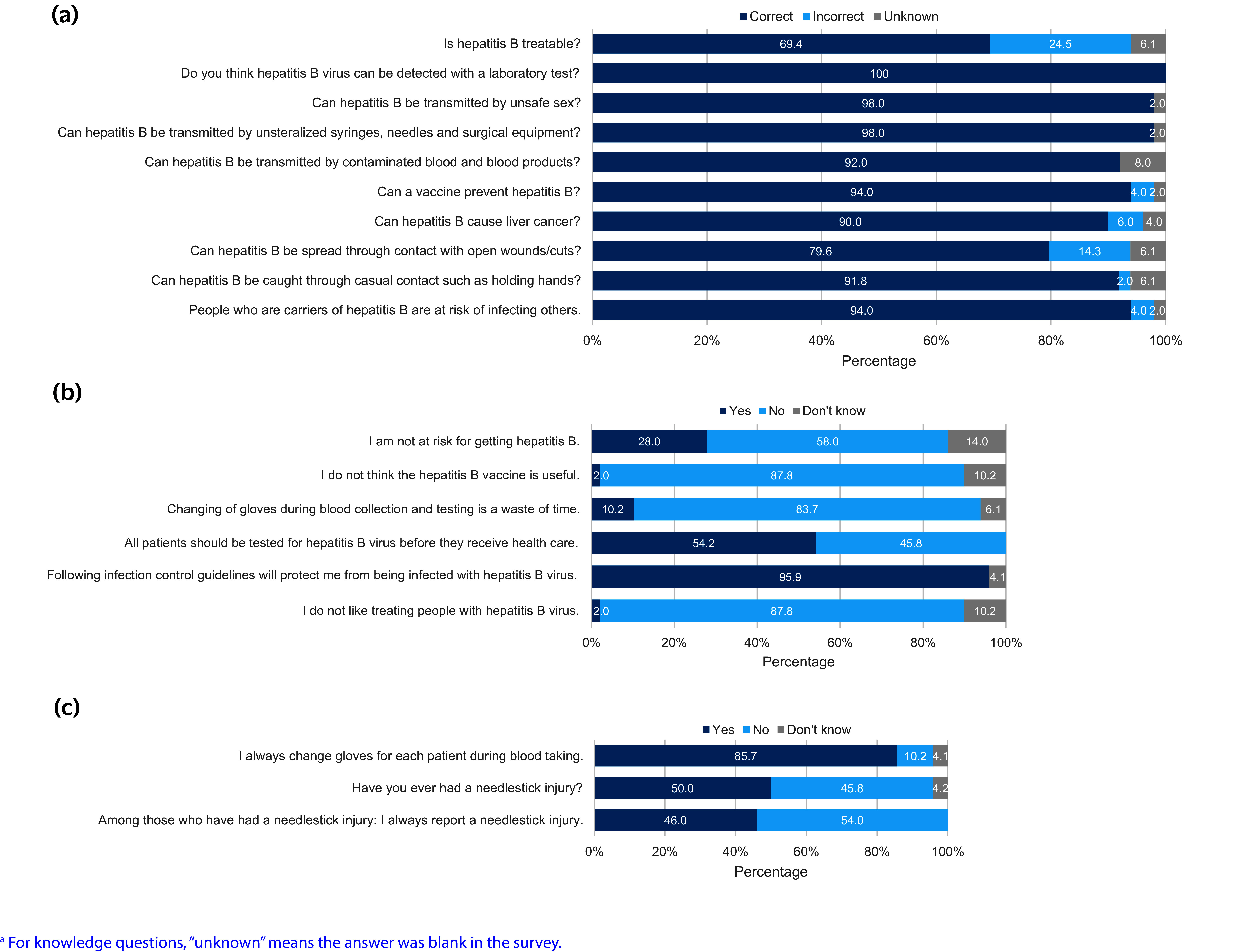
Survey responses to (a) knowledge,^a^ (b) attitudes and (c) clinical practices related to hepatitis B among health-care workers in Vanuatu, 2024 (*N* = 50)

The anonymous survey was open for 2 months (mid-April to mid-June 2024) and was self-administered online using KoboCollect (https://​www​.kobotoolbox​.org/​). It consisted of 30 questions, grouped as follows: demographic characteristics, including occupational group (11 questions); knowledge about hepatitis B (10 questions, **Fig. 1a**); attitudes towards people living with hepatitis B and hepatitis B prevention methods (6 questions, **Fig. 1b**); and clinical practices related to preventing hepatitis B infection (3 questions, **Fig. 1c**). All questions required “yes,” “no” or “don’t know” answers. The questionnaire was based on similar survey tools used in other countries (*17*, *18*) adapted to the Vanuatu context. The survey was available in English and Bislama and was reviewed by the Vanuatu Ministry of Health Viral Hepatitis Unit and the Health Promotions Unit to assess the suitability of the questions and the accuracy of the translation.

[**insert** Figure 1]

### Data and statistical analysis

All questions were optional except for the question confirming informed consent. A knowledge score was calculated for each participant, based on the total number of correct answers (out of 10). If a knowledge question was not answered, it was marked as incorrect. The distribution of the knowledge score was described in terms of a median and interquartile range. The knowledge score was dichotomized to create a binary categorical variable (10 correct answers vs one or more incorrect answers). Due to small sample numbers in some categories (< 5), Fisher’s exact test was used to investigate associations between the dichotomized participant characteristics and knowledge scores.

## Results

A total of 50 participants answered the electronic survey; 33 (66.0%) were born before the introduction of the hepatitis B birth dose, and 36 (73.5%) were female. Most participants worked in hospitals (46.9%) or in non-clinical roles within the Ministry of Health (36.7%), while five participants (10.2%) worked in a community health setting at either a dispensary or health centre. Nearly all participants (93.9%) were born in Vanuatu. Two participants (4.1%) had previously been diagnosed with hepatitis B infection, and neither reported starting treatment (**Table 1**). Respondents were from all six provinces in the country.

**Table 1:** Demographic characteristics among knowledge, attitudes and practice survey participants, Vanuatu, 2024 (*n* = 50)

Characteristic	Value	*n*(%)
**Born before the introduction of the birth dose (***n* **= 50)**	**Yes**	**33 (66.0)**
	**No**	**17 (34.0)**
**Sex (***n* **= 49)**	**Male**	**13 (26.5)**
	**Female**	**36 (73.5)**
**Born in Vanuatu (***n* **= 49)**	**Yes**	**46 (93.9)**
	**No**	**3 (6.1)**
**Province of birth (***n* **= 46)**	**Malampa**	**6 (13.0)**
	**Penama**	**7 (15.2)**
	**Sanma**	**17 (37.0)**
	**Shefa**	**11 (23.9)**
	**Tafea**	**3 (6.5)**
	**Torba**	**2 (4.3)**
**Education (***n* **= 50)**	**Secondary school**	**5 (10.0)**
	**University**	**23 (46.0)**
	**Additional school after university**	**22 (44.0)**
**Type of health-care facility (***n* **= 49)**	**Dispensary**	**2 (4.1)**
	**Health centre**	**3 (6.1)**
	**Hospital**	**23 (46.9)**
	**Ministry of Health**	**18 (36.7)**
	**Private clinic**	**3 (6.1)**
**Type of work (***n* **= 49)**	**Doctor**	**11 (22.4)**
	**Laboratory**	**3 (6.1)**
	**Midwife**	**8 (16.3)**
	**Nurse**	**8 (16.3)**
	**Public health**	**13 (26.5)**
	**Other**	**6 (12.2)**
**Length in job (***n* **= 49)**	** < 1 year**	**1 (2.0)**
	**1–3 years**	**5 (10.2)**
	**3–5 years**	**4 (8.2)**
	**5–10 years**	**12 (24.5)**
	** > 10 years**	**27 (55.1)**
**Previous diagnosis with hepatitis B (***n* **= 49)**	**Yes**	**2 (4.1)**
	**No**	**43 (87.8)**
	**Unsure**	**4 (8.2)**
**For those diagnosed, action taken (***n* **= 2)**	**Treated**	**0 (0)**
	**Never started treatment**	**2 (100)**
**Family history of hepatitis B (***n* **= 49)**	**Yes**	**10 (20.4)**
	**No**	**34 (69.4)**
	**Don’t know**	**5 (10.2)**

### Knowledge

The median knowledge score (out of 10) was 9 (interquartile range: 9–10). The distribution of the scores was highly skewed, with 21 participants scoring 10/10 (100%) and 20 scoring 9/10 (90%). Eight of the 10 questions were answered correctly by over 90% of participants. The two questions with the lowest proportion of correct answers were “Is hepatitis B treatable?” (69.4%) and “Can hepatitis B be spread through contact with open wounds/cuts?” (79.6%) (**Fig. 1a**). Most respondents (*n* = 47, 94.0%) knew that hepatitis B can be prevented with a vaccine, all 50 (100%) knew that hepatitis B virus can be identified with a laboratory test, and 49 (98.0%) knew that hepatitis B virus can be transmitted by unsterilized syringes, needles and surgical equipment, and through unsafe sex.

No significant associations between respondents’ demographic characteristics and level of knowledge were identified (**Table 2**).

**Table 2:** Associations between demographic factors and hepatitis B knowledge score among health-care workers in Vanuatu, 2024 (*n* = 50)

Demographic factor	Knowledge score (dichotomized)		
	≥ 1 incorrect	All correct	Fisher's *P*-value
**Born before the introduction of the birth dose**			
**Yes (***n* **= 33)**	**16**	**17**	**0.075**
**No (***n* **= 17)**	**13**	**4**	**–**
**Sex**			
**Male (***n* **= 13)**	**9**	**4**	**0.348**
**Female (***n* **= 36)**	**19**	**17**	**–**
**Born in Vanuatu**			
**Yes (***n* **= 46)**	**27**	**19**	**0.569**
**No (***n* **= 3)**	**1**	**2**	**–**
**Education^a^**			
**University or less (***n* **= 28)**	**16**	**12**	**1.000**
**Additional education after university (***n* **= 22)**	**13**	**9**	**–**
**Type of health-care facility^b^**			
**Hospital (***n* **= 23)**	**13**	**10**	**0.721**
**Other (***n* **= 26)**	**16**	**10**	**–**
**Type of work^c^**			
**Clinical (***n* **= 27)**	**15**	**12**	**0.771**
**Non-clinical (***n* **= 22)**	**14**	**8**	**–**
**Length in job**			
**≤ 10 years (***n* **= 22)**	**13**	**9**	**1.000**
**> 10 years (***n* **= 27)**	**16**	**11**	**–**
**Known family history**			
**Yes (***n* **= 10)**	**5**	**5**	**0.726**
**No/unknown (***n* **= 39)**	**23**	**16**	**–**

^a^ Education was dichotomized for this analysis.

^b^ Type of health facility was divided into hospital versus other, which included non-clinical Ministry of Health roles and community health workers.

^c^ Clinical category includes doctors, nurses and midwives.

### Attitudes

Most participants (*n* = 43, 87.8%) indicated they thought the hepatitis B vaccine was useful. Most participants (*n* = 47, 95.9%) thought that following IPC guidelines would help to prevent hepatitis B infection in the workplace and that changing gloves was not a waste of time (*n* = 41, 83.7%). Seven participants (14.0%) reported they did not know if they were at risk of hepatitis B infection, and 14 (28.0%) reported they believed that they were not at risk (**Fig. 1b**).

While only one person reported that they did not like treating people with hepatitis B, 26 (54.2%) respondents thought that all patients should be tested for hepatitis B virus before receiving health care.

### Practices

Half of the participants (*n* = 24, 50.0%) reported they had experienced a fingerstick injury, but among them, only 11 (45.8%) said that they always reported the injury. Most participants (*n* = 42, 85.7%) reported that they always change gloves for each patient when taking blood (**Fig. 1c**).

### Discussion

Our results indicate that among HCWs in Vanuatu who responded to our survey, general knowledge about hepatitis B was high. Additionally, there was general acknowledgement of the usefulness of hepatitis B vaccination, which may positively influence vaccine uptake by HCWs. The finding that awareness levels were high is encouraging, particularly because before 2021 response efforts were siloed, with separate units within the Ministry of Health responsible for different activities. However, it is also important to note that participants in this survey were likely a highly engaged, well informed set of HCWs, so knowledge levels may be lower among other HCWs who were less well represented in this sample.

The largest knowledge gap was in participants’ knowledge about hepatitis B treatment, with over 25% unsure whether treatment was available. While there is no cure for hepatitis B, antiviral treatment can reduce the risk of liver disease, including cirrhosis and hepatocellular carcinoma. (*19*, *20*) Tenofovir disoproxil fumarate, an antiretroviral that has been shown to be highly effective, was added to the Vanuatu Essential Drugs List in 2020, but fewer than 200 people are on treatment, despite the high population prevalence of hepatitis B. Two of our study participants had previously been diagnosed with hepatitis B, neither of whom had initiated treatment. If we extrapolate national prevalence estimates to our respondents, we would expect approximately seven people to be living with hepatitis B. Furthermore, if we extrapolate these estimates to the nearly 1000 HCWs in Vanuatu who are eligible for screening according to national guidelines, (*21*) we would expect approximately 100–140 to be living with chronic hepatitis B. This highlights the need for additional screening efforts to identify people living with hepatitis B and link them to care.

The reasons for non-treatment in the two participants with hepatitis B were not explored (as this was beyond the scope of our survey), but our finding is a reflection of low treatment levels nationally. There are many challenges to initiating treatment for hepatitis B in Vanuatu, including a lack of access to laboratory testing, unclear referral pathways and insufficient clinician training, all of which are essential components of treatment services for prescribing and monitoring those on treatment. Additional barriers include low levels of health-seeking behaviour, possibly due to fear of stigma or general trends in access to health services, as well as high costs. While not all study participants would have been directly involved in prescribing, it is important that awareness about the availability and effectiveness of treatment is increased to improve linkage to care, as well as to drive further improvements to health systems to provide this care.

Our survey also revealed relatively low levels of awareness about transmission risk through contact with open wounds and cuts. Limited data are available, but before the roll-out of childhood hepatitis B vaccination in 1989–1990, horizontal transmission was common among children. (*4*, *5*) While the risk of developing chronic hepatitis B infection from exposure as an adult is significantly lower than childhood exposure, (*22*, *23*) transmission through contact with open wounds is a recognized risk for HCWs. Adult exposures can lead to acute hepatitis B infections, which can be severe and carry a risk of liver failure and death. (*24*) The survey indicated high levels of awareness of IPC protocols and guidelines, so perhaps the risk of transmission in health settings is low, but it is important to increase awareness among HCWs of all potential routes of transmission.

Just over 50% of respondents agreed with the statement that all patients should be tested for hepatitis B virus before receiving care. However, it was not possible to infer the reasoning behind participants’ support for universal testing from our study, and this result should be interpreted with caution. While universal testing for hepatitis B is recommended in certain high-prevalence settings and may be cost-effective in some settings, (*25*-*27*) whether it is feasible in the context of Vanuatu needs careful consideration, given the demand for subsequent referrals for treatment and the monitoring that universal testing would generate. Additionally, issues of stigma and discrimination would need to be addressed. Current levels of stigma and discrimination are not well documented in Vanuatu, but globally, internalized and social stigma are commonly reported among people living with chronic hepatitis B. (*28*) In Vanuatu, current practice is that patients may be tested for hepatitis B before surgery, but not before other types of care.

Additional research is required to understand the significance of this apparent high level of interest in universal testing, specifically whether HCWs viewed testing from the perspective of the provision of care for the patient or as a preventive measure to protect themselves. The latter is understandable in the context of HCWs wanting to know patients’ hepatitis B status so they could protect themselves, but this goes against the concept of universal precautions for blood-borne viruses. Further exploration is needed of HCWs’ perspectives on stigma and discrimination around hepatitis B, as well as other blood-borne viruses such as HIV and hepatitis C. While both are currently of low prevalence in Vanuatu, (*1*) they represent an ongoing risk.

Appreciation for the importance of compliance with IPC guidelines among respondents was high, though half of our survey respondents reported fingerstick injuries. More concerning still was the low rate of reporting; among those who had experienced a fingerstick injury, fewer than 50% said that they always reported the event. Globally, hepatitis B is the most commonly acquired infection via needlestick injury. (*29*) While it is likely that most hepatitis B infections in Vanuatu are acquired during childhood, the lower risk of infection in adulthood remains, especially in HCWs. These risks are exacerbated by the absence of an adult vaccine and hepatitis B immunoglobulin in Vanuatu, which are both standard post-exposure prophylaxis for fingerstick injuries in people who are not immunized at birth. Efforts are needed to reduce the risk of fingerstick injury, and to improve reporting among those who experience such injuries and access to post-exposure prophylaxis.

This study has several limitations. First, due to various selection biases, this sample is unlikely to be representative of all Vanuatu’s HCWs. E-mail-based sampling was limited to those 150 workers who were on available e-mail lists, and among those who responded, there was a notable bias towards staff from hospitals and the Ministry of Health. Additionally, because of how the invitation to participate was circulated, it is not possible to estimate the response rate. The e-mail list comprised approximately 150 eligible persons, but the number of eligible persons in the established messaging groups was unknown. Responder bias is also a concern, given that it is probable that participants may have been more aware of, or more interested in, hepatitis B than those who did not respond. Finally, the sample size was small and therefore reduced the likelihood of observing statistically significant associations.

### Conclusion

These findings have potential implications for public health policy and practice in Vanuatu, particularly in relation to protocols for the introduction of adult and HCW immunization against hepatitis B, as well as priorities for continuing medical education and future research. Further research is needed to explore the knowledge, attitudes and practices of HCWs working in community-based facilities outside the main cities in Vanuatu. There is also a need to better understand the stigma and discrimination associated with hepatitis B. Despite its limitations, this study has provided useful insights that will help guide the development of HCW educational sessions ahead of the planned roll-out of the HCW vaccination campaign.
